# Hypoxia-related long noncoding RNAs are associated with varicocele-related male infertility

**DOI:** 10.1371/journal.pone.0232357

**Published:** 2020-04-30

**Authors:** Nafiseh Sanei Ata-abadi, Seyed Javad Mowla, Fatemeh Aboutalebi, Kianoush Dormiani, Abbas Kiani-Esfahani, Marziyeh Tavalaee, Mohammad Hossein Nasr-Esfahani

**Affiliations:** 1 Department of Molecular Genetics, Faculty of Biological Sciences, Tarbiat Modares University, Tehran, Iran; 2 Department of Molecular Biotechnology, Cell Science Research Center, Royan Institute for Biotechnology, ACECR, Isfahan, Iran; 3 Department of Cellular Biotechnology, Cell Science Research Center, Royan Institute for Biotechnology, ACECR, Isfahan, Iran; 4 Department of Reproductive Biotechnology, Reproductive Biomedicine Research Center, Royan Institute for Biotechnology, ACECR, Isfahan, Iran; 5 Isfahan Fertility and Infertility Center, Isfahan, Iran; Universite Clermont Auvergne, FRANCE

## Abstract

One of the main molecular causes that contributes to varicocele-related male infertility is excess production of reactive oxygen species (ROS). It is believed that hypoxia is an important stimulator of ROS in this condition. Recently, the significant roles of long non-coding RNAs (lncRNAs) in hypoxia response have emerged. Despite the investigation of hypoxia, there is scant information about the role of hypoxia-responding lncRNAs in varicocele-related male infertility. In the present study, we deduced eight hypoxia-responding lncRNAs based on high-throughput RNA sequencing data from two Gene Expression Omnibus (GEO) datasets. We used qRT-PCR to assess the expression levels of some of these lncRNAs in 42 ejaculated spermatozoa samples from 25 infertile men with varicocele and 17 fertile men as controls. We identified significant increases in expression levels of hypoxia-related lncRNAs, *MIR210HG* and *MLLT4-AS1* in ejaculated spermatozoa of infertile men with varicocele. These lncRNAs also showed significant positive correlations with ROS levels and meaningful negative correlations with sperm parameters (count and motility). Besides, in silico studies identified several hypoxia response elements (HREs) within selected lncRNAs promoters. Delineation of hypoxia-related lncRNAs in varicocele-related infertility provides a valuable insight into male infertility.

## Introduction

Varicocele is defined as abnormal tortuosity, dilatation, and elongation of the pampiniform plexus veins in spermatic cord. Approximately 40% of infertile men are diagnosed with varicocele; thus, it is a leading cause of male infertility [1]. Varicocele is responsible for about 19%–41% of primary male infertility and approximately 80% of secondary male infertility. Oxidative stress, hypoxia, heat stress, back flow of metabolites, and hormonal disturbances are possible etiologies for varicocele mediated male infertility. However, the exact mechanism of varicocele related male infertility has not been elucidated [2]. Therefore, identification of biological markers related to this issue is crucial.

Recently, long non-coding RNAs (lncRNAs), which are non-protein coding RNAs above 200 nucleotides in length, are postulated to be ideal diagnostic biomarkers and therapeutic targets that serve important regulatory functions in many biological processes [3]. LncRNAs in human sperm were initially characterized by Sendler et al., who used RNA sequencing technique [4]. Although there are many lncRNAs in human sperm, their expressions and roles in varicocele-related male infertility remain to be determined [5]. Recently, Zhao et al., assessed the expression level and function of an oxidative stress-related lncRNA, named growth arrested DNA-damage inducible gene 7 (*GADD7*), in spermatozoa of individuals with varicocele. They demonstrated that this lncRNA markedly up-regulated in the varicocele group compared to the healthy control group. Furthermore, they indicated that *GADD7* overexpression suppressed cell proliferation and induced apoptotic cell death in mouse spermatocyte-derived cell lines, GC-1 and GC-2. They believed that regulation of cell proliferation and apoptosis by this lncRNA resulted in reduction of sperm counts in individuals with varicocele [6].

Hypoxia is one of the most important mediators of varicocele-related male infertility. A wealth of independent studies have reported adverse effects of hypoxia on male infertility and its relationship with sperm count and motility. Emerging data suggest that intracellular hypoxia is the main source of oxidative stress in varicocele [7–10]. The main mediator of hypoxia is a family of transcription factors known as hypoxia inducible factors (HIFs). HIF is a heterodimer composed of two subunits, HIF-1α and HIF-1β. Under hypoxic conditions, this transcription factor regulates the expression of several hypoxic response genes [11]. In varicocele, hypoxia leads to an elevation in HIF-1α expression and subsequently vascular endothelial growth factor (VEGF), which ultimately regulates cellular response to hypoxia [12, 13]. The pivotal roles of lncRNAs in hypoxia-driven diseases have been recognized. LncRNAs respond to hypoxia via HIF-mediated direct or indirect interactions and are involved in regulation of hypoxic gene expression at the genomic, transcriptional, and post-transcriptional levels. In HIF-mediated direct regulation, lncRNA transcription is directly regulated by HIF through hypoxia response elements (HREs) within their promoters. Transcriptional activation of *HOTAIR*, *MALAT1*, *H19*, *ANRIL*, and *NEAT1* are mediated through HRE under hypoxic conditions. Indirect transactivation of lncRNAs seems to be attained through HIF-1α mediated epigenetic mechanisms [14].

In the present study, we used previous high-throughput RNA sequencing data from the Gene Expression Omnibus (GEO) dataset to identify a set of lncRNAs that participate in the hypoxia response. Real-time qPCR analysis showed that some of these lncRNAs had significantly increased expression in ejaculated spermatozoa from infertile men with varicocele compared with spermatozoa from fertile men. We conducted an in silico study to predict the presence of HRE within their regulatory sequences. To the best of our knowledge, this is the first report that investigated hypoxia modulated lncRNAs in varicocele-related male infertility. Our finding may unveil novel diagnostic biomarkers and/or therapeutic targets for male infertility.

## Materials and methods

### Patients

This case-control study included of 25 individuals with grade II or III varicocele who referred to the Isfahan Fertility and Infertility Center (IFIC), Isfahan, Iran for infertility treatment and 17 healthy donors with normal semen parameters and no clinical presentation of varicocele who referred to the IFIC for family balancing. We excluded men with leucocytospermia, azoospermia, seminal sperm antibodies, testicular size discrepancy, fever within 90 days prior to seminal analysis, cancer, abnormal hormonal profiles, recurrent varicocele, grade I varicocele, urogenital infections, previous history of scrotal trauma or surgery, drug consumption, excessive alcohol, or occupational exposure. Informed written consents were obtained from all participants. The present study received the approval of the Institutional Review Board of Royan Institute (ID. No. IR.ACER.ROYAN.REC.1397.272).

### Semen samples

Semen samples were collected after 3–5 days of sexual abstinence and allowed to liquefy at room temperature for 30 min. Semen analysis was performed on one portion of the samples according to World Health Organization [15] criteria for assessment of sperm concentration, morphology, motility and vitality. The remainder of the semen samples were washed twice in phosphate buffer saline (PBS; pH 7.4) and used for RNA extraction.

### Assessment of reactive oxygen species (ROS) production using DCFH-DA probe

We assessed the ROS levels of the spermatozoa samples with a cell-permeable stain, 2', 7'-dichlorodihydrofluorescein diacetate (DCFH-DA; Sigma-Aldrich, MO, USA). DCFH-DA was added to the sperm suspension (1 × 10^6^ sperm ml^−1^ in PBS) at a concentration of 0.5 μM, incubated at 37°C for 30 min and analyzed by a fluorescence detector-1 (FL-1) with a 530/30 nm band pass filter in a FACSCalibur flow cytometer (Becton Dickinson, San Jose, CA, USA). CellQuest Pro and WinMDI 2.9 software were used for data analysis. Every test sample was normalized against an unstained control sample. Data were expressed as the percentage of fluorescent positive spermatozoa.

### Long non-coding RNA (lncRNA) selection

Whole transcriptome RNA-sequencing data were retrieved from the GEO datasets, GSE76743 (Platform: GPL11154; Illumina HiSeq 2000) [16] and GSE70544 (Platform: GPL11154; Illumina HiSeq 2000) [17], to identify differentially expressed lncRNAs (DE lncRNAs) that respond to hypoxia in two different cell types including human umbilical vein endothelial cells (HUVECs) and human proximal tubular epithelial cells (PTECs), respectively. The processed data of each dataset was used to identify DE lncRNAs between hypoxia-treated samples and their controls with a p-value < 0.05. The overlapping DE lncRNAs between two datasets were determined using the Visual Basic for Applications (VBA) programming language.

### RNA extraction and cDNA synthesis

Total RNA was isolated from sperm pellets using TRIzol reagent (Invitrogen, USA) according to the manufacturer’s supplied instructions. The quality of extracted RNA was evaluated by agarose electrophoresis and its quantity determined by measuring absorbance at 260 nm using a NanoDrop spectrophotometer (Nanodrop 1000, Thermo Scientific, USA). Total RNA was treated with RNase-free DNaseI (Thermo Scientific, USA).Synthesis of first strand cDNA was performed using a PrimeScript II 1st Strand cDNA Synthesis Kit (TaKaRa, Japan).

### Real-time qPCR

For real-time qPCR, cDNA templates were mixed with SYBR Premix Ex TaqII (TaKaRa, Japan) in a final volume of 10 μl. All reactions were carried out in triplicate using specific primers. The PCR experiment was performed during three steps with an initial denaturation at 95°C for 30 s and 40 cycles that consisted of a denaturation step at 95°C for 5 s, an annealing step at specific annealing temperature for 10 s, and an extension step for 30 s at 72°C. A Step One Plus Real-Time PCR thermal cycler (Applied Biosystems, USA) was utilized for performing real-time qPCR.

Real time specific primers for each of the lncRNAs were designed using Beacon designer 8.13 (Premier Biosoft, USA) and rechecked with Oligo 7.56 software (Molecular Biology Insights, USA) for secondary structures and target specificity. Designed primers are listed in Table 1. The expression levels of the lncRNAs were normalized by expression of the housekeeping gene, *GAPDH*. Relative expression was calculated using the ΔΔCt method [18, 19].

**Table 1:** Primer pairs used in this study.

Primer	Sequence (5ˊ-3ˊ)	Tm (˚C)	GC%
F-*MIR210HG*	GGACCTGCACCTGAGAAAG	55	57.9
R-*MIR210HG*	CACAGTAAGTCGCCGTC	54	58.8
F-*MLLT4-AS1*	TTCACTGAACCTCAACTG	52	44.4
R-*MLLT4-AS1*	TGCAAACTCATTCTTCAAGAG	51	38.1
F-*RP11-540A21*.*2*	CCCACCCTCACTTCTAAATC	56	50
R-*RP11-540A21*.*2*	CTGAATCTCACGGACATCTTG	56	47.6
F-*GAPDH*	CCACTCCTCCACCTTTGACG	60	60
R-*GAPDH*	CCACCACCCTGTTGCTGTAG	62	60

F, forward primer; R, reverse primer; Tm, melting temperature.

### *In silico* analysis of hypoxia responsive elements (HREs)

We used the Genomatix MatInspector database (http://www.genomatix.de) [20] to test the existence of putative HREs in the predicted promoter of selected hypoxia-related lncRNAs. MatInspector is a software tool that utilizes a large library of matrix descriptions for transcription factor binding sites to scans nucleotide sequences for matches. To achieve this end, MatInspector calculates a “matrix similarity score” which reaches 1 only if matches occur completely. Usually, a matrix similarity of 0.85 will imply a good match for most trials. Optimized length of promoters in this database is nearly 1,000 bp upstream of the first transcription start site (TSS) and 100 bp downstream of the last TSS [20]. Prediction of putative HREs in our study, was carried out with a matrix similarity of 0.85.

### Statistical analysis

Data normality was evaluated using the Kolmogorov-Smirnov Z test. A standard two-tailed student’s t-test was used to compare the mean expression levels of our reference and target genes between the varicocele and control groups. Pearson’s coefficient was used for correlation assay in this study. The aforementioned statistical analyses were carried out using Graph Pad Prism (v6; GraphPad software) and SPSS 20 software (SPSS, Chicago, IL, USA). For all analyses, p-values less than 0.05 were considered to be statistically significant.

## Results

### Semen analysis

Table 2 lists the results of semen analyses of the varicocele and fertile groups. No differences were noted in the mean age and volume between two groups. The mean sperm count and percentage of sperm motility both significantly decreased. There was a significant increase in the percentage of abnormal sperm morphology in infertile men with varicocele compared to fertile men, which indicated impaired spermatogenesis in the varicocele group.

**Table 2:** Descriptive of semen parameters.

Parameters	Healthy control (n = 17)	Varicocele (n = 25)	P-value
Age (years)	35.12 ± 1.286	32.80 ± 0.7874	0.1117
Volume (ml)	3.924 ± 0.3088	3.528 ± 0.2616	0.3368
Concentration (10^6^/ml)	90.88 ± 6.741	40.16 ± 4.123	<0.0001
Motility (%)	60.00 ± 1.917	41.76 ± 2.650	<0.0001
Abnormal morphology (%)	96.24 ± 0.2353	97.40 ± 0.2582	0.0030

### Reactive oxygen species (ROS) assessment

**Fig 1:**
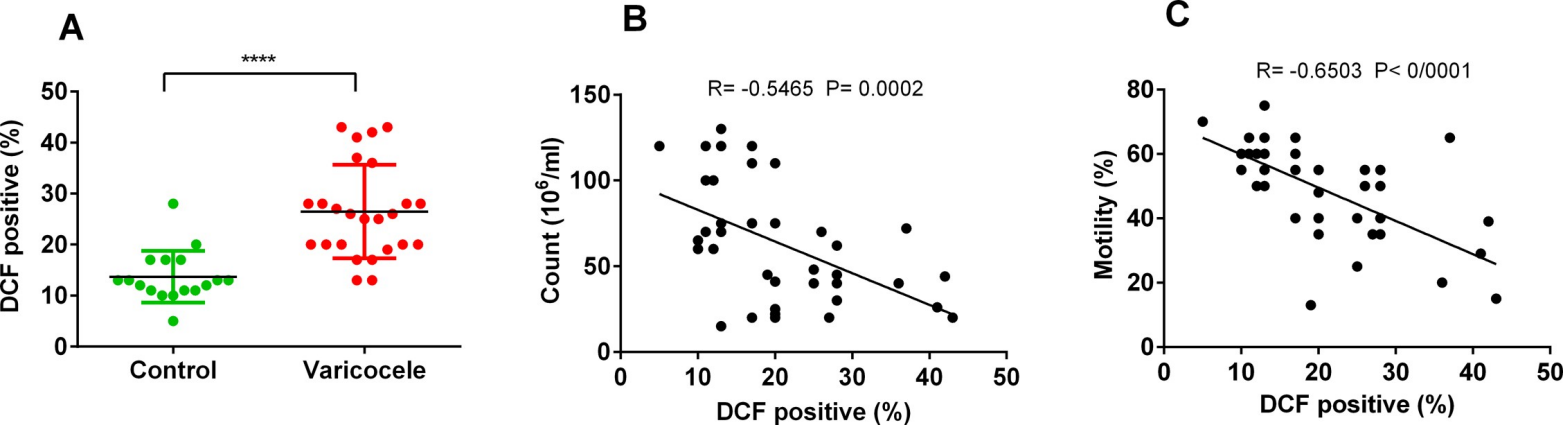
Determination of reactive oxygen species (ROS) levels in the varicocele and control groups and its relationship with sperm count and motility. (A) Reactive oxygen species (ROS) levels in the varicocele and control groups assessed by DCF-DA staining (****P<0.0001 using the unpaired t-test). ROS levels had a negative correlation with sperm count (B) and motility (C). Pearson’s coefficient was used for correlation assay.

ROS levels were assessed in sperm of varicocele and fertile individuals using DCFH-DA staining. There was a significant elevation in the percentage of DCF positive sperm in infertile men with varicocele compared to fertile men, which indicated a high level of oxidative stress in the varicocele state (Fig 1A). We assessed the correlation between ROS levels with semen parameters. As indicated in Fig 1B and 1C, there were significant negative correlations between ROS levels with sperm count and motility.

### Putative long non-coding RNAs (lncRNAs) associated with hypoxia

**Fig 2:**
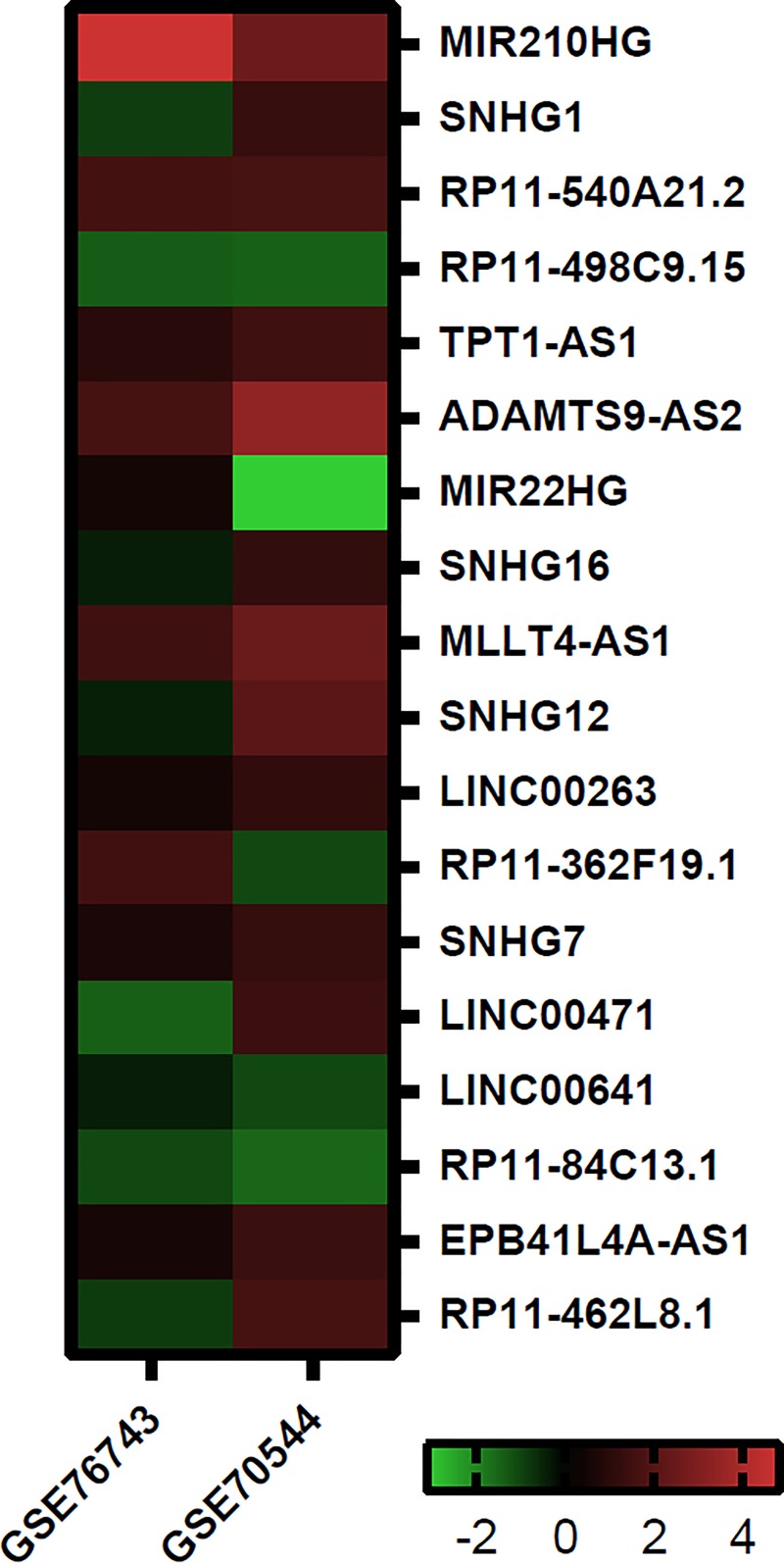
Heat maps of overlapping differentially expressed long non-coding RNA (lncRNA) responding to hypoxia in two Gene Expression Omnibus (GEO) datasets.

Since hypoxia is one of the inducers of ROS and a potential mechanism in varicocele-related male infertility, we hypothesized that the lncRNAs stimulated by hypoxia may have a relationship with varicocele. Therefore, we sought to identify if any of the lncRNAs related with hypoxia are also involved in varicocele-related male infertility. So we selected two GEO datasets, (GSE70544 and GSE76743) that have been identified hypoxia-related lncRNAs in the response to hypoxia induction in two different cell types; one of them (GSE76743) in HUVECs and the other (GSE70544) in PTECs. Then, we compared the results of two GEO datasets, (GSE70544 and GSE76743) and identified 18 lncRNAs that responded to hypoxia in both studies (S1–S3 Tables, Fig 2). These overlapping lncRNAs could potentially respond to hypoxia in other cell types such as testicular cells. We noted that 8 out of 18 lncRNAs showed one orientation, so that their expressions either concurrently increased or decreased following induction of hypoxia. LncRNAs *MIR210HG*, *MLLT4-AS1*, *RP11-540A21*.*2*, *TPT1-AS1*, and *ADAMTS9-AS2* were up-regulated, whereas *RP11-498C9*.*15*, *LINC00641*, and *RP11-84C13*.*1* were down-regulated. Finally, we selected three lncRNAs—*MIR210HG* (*ENSG00000247095*), *MLLT4-AS1* (*ENSG00000198221, AFDN-AS1*), and *RP11-540A21*.*2* (*ENSG00000245522*, *LOC440028*) for further analysis.

### Significant differences in expression levels of hypoxia-responding long non-coding RNAs (lncRNAs) in infertile men with varicocele

In order to confirm the differential expression of hypoxia responding lncRNAs in varicocele, we evaluated the expression levels of three selected lncRNAs in spermatozoa of 26 infertile men with varicocele and 17 controls.

**Fig 3:**
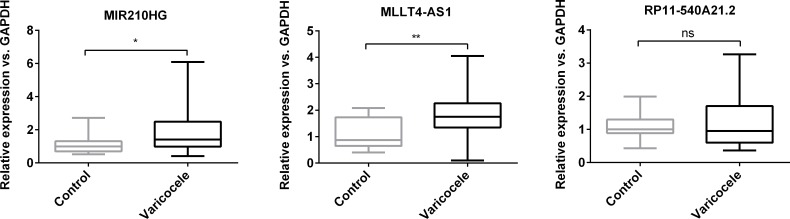
Long non-coding RNA (lncRNA) expressions in the varicocele and control groups. Boxplots of the expression levels of lncRNAs *MIR210HG*, *MLLT4-AS1*, and *RP11-540A21*.*2* in the ejaculated spermatozoa from the varicocele group compared to the control groups (P = 0.0307 for *MIR210HG*; P = 0.0061 for *MLLT4-AS1*; P = 0.4111 for *RP11-540A21*.*2*; two-tailed sample t-test; ns: not significant). Levels of expression were normalized relative to *GAPDH*.

Real-time qPCR data analysis showed significant elevations in *MIR210HG* (P = 0.0307) and *MLLT4-AS1* (P = 0.0061) expressions in sperm of infertile men with varicocele compared to the control group. *RP11-540A21*.*2* was also up-regulated in varicocele patients; however, this up-regulation was not significant for our samples (P = 0.4111; Fig 3).

### Expression levels of hypoxia responding long non-coding RNAs (lncRNAs) showed significant correlations with reactive oxygen species (ROS) levels and sperm parameters

**Fig 4:**
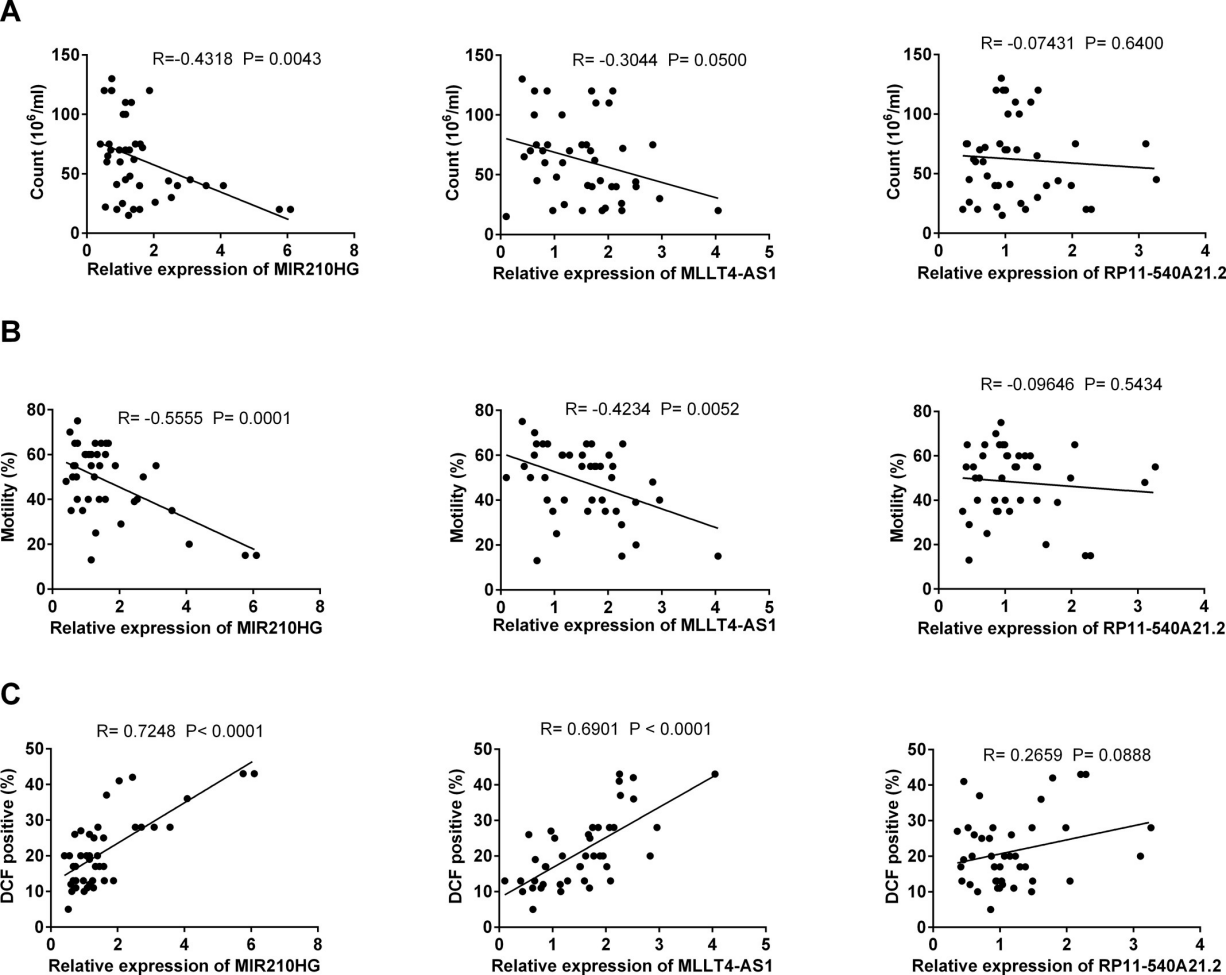
Correlation analysis between the expression levels of long non-coding RNAs (lncRNAs) with sperm parameters and reactive oxygen species (ROS) levels. **(A)** There are meaningful negative correlations between the expression levels of *MIR210HG* and *MLLT4-AS1* with sperm count. **(B)** Expression levels of *MIR210HG* and *MLLT4-AS1*, but not *RP11-540A21*.*2*, show a significant negative correlation with sperm motility. **(C)** Significant positive correlation exist between the expression levels of *MIR210HG* and *MLLT4-AS1*, but not *RP11-540A21*.*2*, with reactive oxygen species (ROS) levels. Pearson’s coefficient correlation was used for correlation analysis.

In order to confirm that the selected lncRNAs specifically participated in varicocele-related male infertility, we examined the correlation of their expression levels with sperm parameters and ROS levels. As expected, *MIR210HG* and *MLLT4-AS1*, but not *RP11-540A21*.*2*, showed meaningful negative correlations with sperm count and motility (Fig 4A and 4B) and positive correlations with ROS levels in our spermatozoa samples (Fig 4C).

### Presence of hypoxia response elements (HRE) in the predicted promoters of hypoxic long non-coding RNAs (lncRNAs)

**Fig 5:**
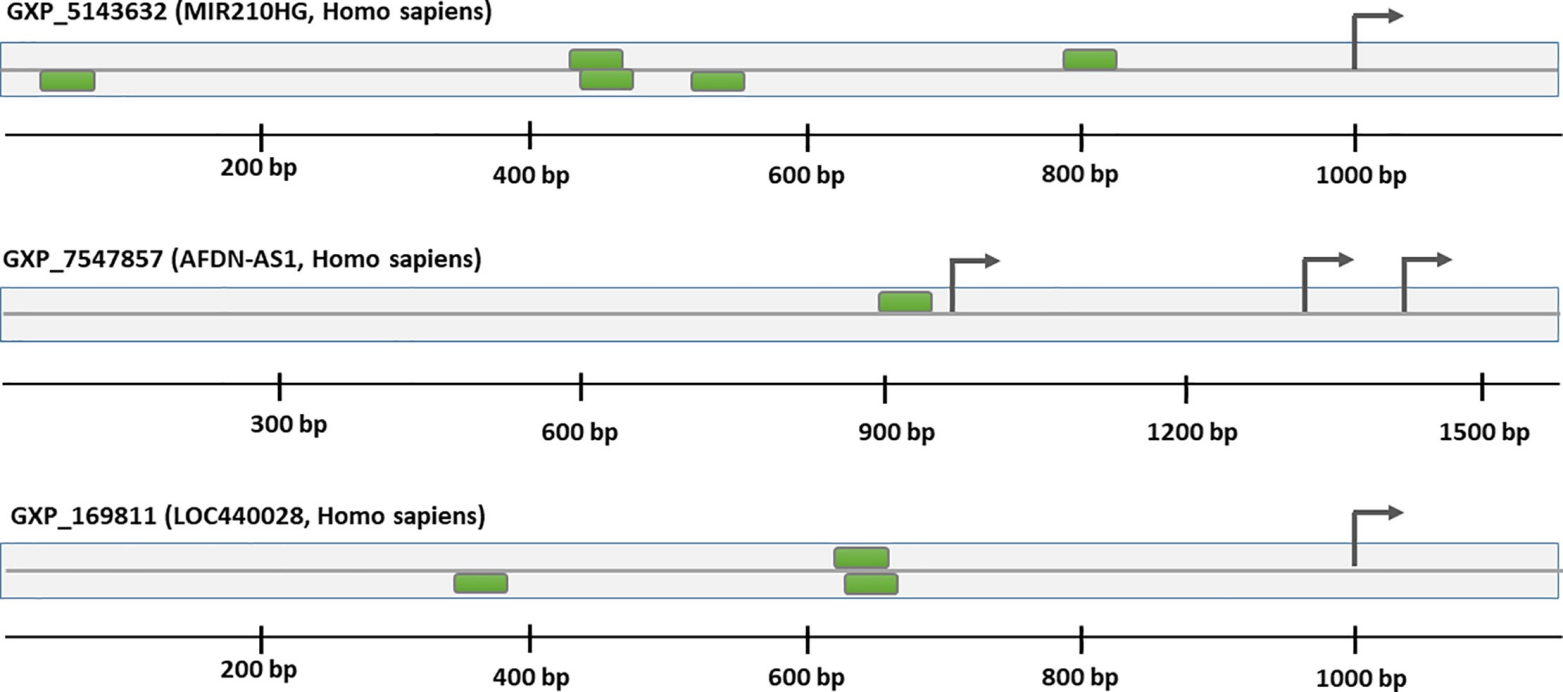
Presence of hypoxia response elements (HREs) in the predicted promoters of long non-coding RNAs (lncRNAs). All three lncRNAs harbored the hypoxia response elements (HRE) within their promoters that identified by the Genomatix MatInspector database. Genomatix optimized length for the promoters is nearly 1,000 bp upstream of the first TSS and 100 bp downstream of the last TSS. Green boxes indicated HREs in the predicted promoter sequences on both orientations (“+” or upper strand or 5'—>3՛; “–” or lower strand or 3'—>5՛). Black arrows represented transcription start sites (TSS). Three TSS on the *MLLT4-AS1* (*AFDN-AS1*) promoter indicated three alternative transcripts identified by Genomatix MatInspector database.

We scanned the area 1 kb upstream of the transcription start site of the hypoxia-related lncRNAs *MIR210HG*, *MLLT4-AS1*, and *RP11-540A21*.*2* using the Genomatix MatInspector database for matches to HIF-1α binding matrices. This analysis revealed that one or more HREs were located in the predicted promoters of *MIR210HG*, *MLLT4-AS1* (*AFDN-AS1*), and *RP11-540A21*.*2* (*LOC440028*) (Fig 5). These data suggested a possibly direct regulation of these predicted hypoxia responding lncRNAs by the HIF-1 pathway.

## Discussion

It is well accepted that varicocele can lead to impaired spermatogenesis and it is one of the most important cause of male infertility via induction of hyperthermia, oxidative stress, and hypoxia. There have been extensive efforts in identifying mechanisms of varicocele-related male infertility. However the exact molecular pathological process remains to be elucidated. Growing evidences indicate that lncRNAs are involved in regulation of hypoxia responses, one of the elements that contribute to varicocele-related male infertility [12–14].

In the present study, we sought to identify hypoxia response lncRNAs by reanalyzing two separate high throughput studies from GEO datasets (GSE70544 and GSE76743) [16, 17]. The results showed that several shared lncRNAs significantly responded to hypoxia in one orientation with simultaneous up-regulation or down-regulation. Finally, we selected three hypoxia-related lncRNAs (*MIR210HG*, *MLLT4-AS1*, and *RP11-540A21*.*2*) for experimental validation by real-time qPCR in spermatozoa of infertile men with varicocele and fertile men. Among these three lncRNAs, *MIR210HG* and *MLLT4-AS1* significantly up-regulated in spermatozoa of infertile men with varicocele compared to spermatozoa from the normal fertile group. However *RP11-540A21*.*2* expression did not show any significant difference between these two groups. The insignificant expression level of *RP11-540A21*.*2* could be due to the higher expression variation of this lncRNA relative to others in spermatozoa, which might be solved with a larger sample size.

Interestingly, the relative expression levels of *MIR210HG* and *MLLT4-AS1* had a positive correlation with ROS level, which showed potential role of hypoxia-related lncRNAs during high levels of ROS production in infertile men with varicocele. Moreover, the significant negative correlations of *MIR210HG* and *MLLT4-AS1* with sperm count and motility reflected the relationship of these lncRNAs with sperm impairment in varicocele-related male infertility.

The transcription factor HIF-1, as a master regulator of O2 homeostasis, has an important role in regulation of hypoxia response genes. The HIF-1 binding sites across these selected lncRNAs were recognized using the Genomatix MatInspector database. Remarkably, for all three selected lncRNAs (*MIR210HG*, *MLLT4-AS1*, and *RP11-540A21*.*2*), HIF-1 occupancy was found within their predicted promoters. These results showed that HIF-1 might directly regulate transcription of these lncRNAs through HREs within their promoters.

As mature spermatozoa is considered to be transcriptionally silent, the RNA content of mature spermatozoa could be the residue of RNAs transcript during spermatogenesis. The confirmation of this claim is the fact that mRNAs seen in spermatozoa exist in human testis, too [21]. Therefore, potentially transcription elevation of selected lncRNAs through HIF-1 occurs in spermatogenesis stages in testis of infertile men with varicocele in response to hypoxia. However, further studies are needed to approve this hypothesis.

*MIR210HG* is the host gene of miR-210, a well-known hypoxia microRNA, induced by HIF-1α [17]. *MIR210HG* was validated in hypoxic HUVECs and human PTECs as hypoxia responding lncRNAs [16, 17]. In addition, this lncRNA is a proposed potential tumor biomarker for glioma [22], colorectal adenocarcinoma [23], osteosarcoma [24], and pancreatic cancer [25]. In the present study, we reported that *MIR210HG* can be a potential biomarker of varicocele-related male infertility.

*MLLT4-AS1* was found to be down-regulated in gastric cancer and its lower expression was reported to be associated with advanced Tumor-Node-Metastasis stage and lymph node metastasis in this cancer [26]. *MLLT4-AS1* was reported to be one of the top DE lncRNAs that expresses between the oral cavity and oropharyngeal squamous cell carcinoma (OSCC) tissues and healthy oral mucosa [27]. While a relationship between *MIR210HG* and hypoxia has been determined in previous studies [16, 17], this paper is the first study that revealed a relationship between *MLLT4-AS1*and hypoxia.

Although the role of *RP11-540A21*.*2* is poorly understood, a handful of studies evaluated its expression and showed that it had a significant relationship with human head and neck squamous cell carcinoma [28] and primary aldosteronism [29]. In this study, the expression level of *RP11-540A21*.*2* was elevated, but not statistically significant, between the varicocele and control groups.

In conclusion, we observed an association between the expression levels of hypoxia responding lncRNAs with varicocele-related male infertility and showed their expression levels have a significant correlation with ROS levels in this condition. Our study might help to improve our understanding of the mechanisms in varicocele-related male infertility and provide diagnostic biomarkers and therapeutics targets.

## Supporting information

S1 Table Differentially expressed long non-coding RNAs (DE lncRNAs) that responded to hypoxia in human umbilical vein endothelial cells (HUVECs) retrieved from the GSE76743 dataset. (XLSX)

S2 Table Differentially expressed (DE) protein coding transcripts and long non-coding RNAs (lncRNAs) that responded to hypoxia in proximal tubular epithelial cells (PTECs) retrieved from the GSE70544 dataset. (XLSM)

S3 Table Overlapping differentially expressed long non-coding RNAs (DE lncRNAs) between the GSE76743 and GSE70544 datasets. The ensemble ID, chromosomal location, fold changes and P-values were presented for selected long non-coding RNAs (lncRNAs).
(XLSM)
